# Simulation and Validation of Optimized PID Controller in AGV (Automated Guided Vehicles) Model Using PSO and BAS Algorithms

**DOI:** 10.1155/2022/7799654

**Published:** 2022-11-14

**Authors:** Ata Jahangir Moshayedi, Jinsong Li, Nima Sina, Xi Chen, Liefa Liao, Mehdi Gheisari, Xiaoyun Xie

**Affiliations:** ^1^School of Information Engineering, Jiangxi University of Science and Technology, No. 86, Hongqi Ave, Ganzhou 341000, Jiangxi, China; ^2^Department of Applied Mathematics and Computer Science, Technical University of Denmark Kgs, Lyngby, Denmark; ^3^Department of Mechanical Engineering, Iowa State University, Ames, IA, USA; ^4^Department of Computer Science and Technology, Harbin Institute of Technology, Shenzhen, China; ^5^Department of Cognitive Computing, Institute of Computer Science and Engineering, Saveetha School of Engineering, Saveetha Institute of Medical and Technical Sciences, Chennai, India; ^6^School of Electronic Information Engineering, Gannan University of Science and Technology, Ganzhou, Jiangxi, China

## Abstract

Automated guided vehicles (AGVs) are popular subsets of robots that come in various shapes and sizes. The group's use in the industry ranges from applications for carrying pallets, carts, and utensils to helping the elderly or transporting medicine to hospitals. Even recently, they have been used in libraries to carry books on shelves. The main part of an AGV includes its body, motor, driver, processor, and sensors, which are more or less the same in all types of AGVs, and addons vary depending on the application and the work environment. The part that affects AGV performance is the control strategy, to which researchers have shown different approaches. Using various techniques and simulations to obtain a model is the key and can help to improve and evaluate the performance of the strategy of the robot. In this study, based on the actual design of the AGV system, all data and components are described, and the simulation is performed in MATLAB software. Then, for controlling the platform based on the PID controller tuning, four methods of Ziegler Nichols, empirical, Particle Swarm Optimization (PSO), and Beetle Antennae Searching (BAS) (optimizer) are discussed, and the results are compared in the four paths including the circle, ellipse, Spiral and 8-shaped paths by observing and testing the tuned PID parameters. Finally, a series of subsequent experiences were carried out in CoppeliaSim (VREP) as a famous robot simulator to overcome the environmental constraints for the same paths that were used in Matlab based on the extracted PID values. Based on the results, the empirical methods, PSO, and BAS errors are very close together. But in general, the BAS algorithm is the fastest, and the PSO had better performance. In general, the maximum error is linked to the path of 8 shapes and the minimum is related to circle shape one. Finally, the analysis of results in different paths in both simulators shows the same results. Therefore, concerning the limited test on the real platform and using the PID coefficients obtained from the simulation shows the model's ability for the researchers in robotic research.

## 1. Introduction

Automated Guided Vehicles (AGVs) play a pivotal role in robotics and have become an indispensable part of it. Based on history, AGVs introduced in 1953 are known as comparatively older inventions, but regardless of their age, the increasing applications show the increased demand in the industry and are being improved by AI day by day [1]. These improvements open up an opportunity for researchers to investigate the limitations that AGV robots may bring. The AGVs are used in various applications, and recently due to COVID-19 restrictions, AGVs' demand is increased in various applications to provide better service and assistance in hotels, hospitals, restaurants, assist patients, etc., during the lockdowns around the world [2]. AGVs consist of three main parts: the frame and its installed device, models and sizes, and the navigation and actuator systems. Based on their application and role as service robots, these robots can be categorized in various shapes and structures, from dual-wheel to worm shapes [3]. But other than the shape, the control section is known as one of the main issues investigated by various researchers over the years. Reviewing published research about the AGV controlling part shows,Kodagoda et al. showed a fuzzy logic control (FLC) with uncoupled vertical and horizontal control for a golf car as the AGV. This AGV has four wheels, with the steering control system, which is driven by a permanent magnet DC motor besides, the active deceleration of AGV is to drive the stepping motor to control the cable connected to the brake golf car pedal. The encoder calculates the speed of AGV on four wheels, and the encoder obtains the steering angle and angular speed on the steering shaft. The longitudinal control is realized by two uncoupled fuzzy drive controllers and fuzzy brake controllers, and the lateral controller is also obtained by fuzzy logic. By comparing the proposed FLC scheme with the traditional PID scheme, the paper concluded that the proposed FLC have better performance in tracking, smaller steady-state error, and powerful in load changing, coupling effects, operating point changes, and road grade changes [4]. Suárez et al. designed a method to determine the Ackerman steering system's longitudinal and steering system model-based AGV. They proposed the longitudinal model based on the speed step response and obtained the steering system model by simulating the behaviour of the PID controller, the motor, and the steering system load with MATLAB. The fuzzy controller, pure pursuit controller, ɛ-Controller with PI regulator, and ɛ-Controller with the PI regulator are used and was tested on the semicircular path with a radius of 25 meters. The result shows that the fuzzy controller, pure pursuit controller performed worst, and the ɛ-Controller with PI regulator and ɛ-Controller with the PI regulator is robust, and almost both controllers worked the same [5]. Chen et al. proposed PID controllers and fuzzy PID controllers for electric vehicles driven by two-wheel hub motors. The AGV simulation was built on two rear-wheel hub motors that drove eleven degrees of freedom and two models. They proposed adjusting the traction force acting on the two drive wheels according to the slip rate and yaw rate to improve the handling and stability of the vehicle. Through the simulation experiment in MATLAB/Simulink environment, comparing PID controller, fuzzy PID controller, and without a controller, they found that PID controller and fuzzy PID controller can improve the control stability of the vehicle. However, it does not affect the vertical movement of the vehicle [6]. Faber Archila and Becker developed an electric vehicle with four wheels driven by two hub DC motors mounted on the front axle and the control system. The control system consists of two levels. The motor controller is responsible for running the motor in the appropriate model at the low level to generate the required motor torque. The torque coordination controller can correctly coordinate the motor torque at a high level and provide stability and consistent steering performance during the control saturation period. The Adams software is used for simulation as the simulator, and the current control adopts the PI controller. The results show that the control system can successfully realize the vehicle's drive-by-wire, steer-by-wire, and power steering functions [7]. Gupta et al. proposed a trajectory tracking system based on a tricycle AGV that involves a front driving wheel and two passive rear wheels. The trajectory tracking algorithm is used as the pure tracking algorithm based on curvature calculation which is worked based on forward-looking distance as the main used parameter calculating the arc. Besides, in their research, the laser and receiver SICK equipment (AUTO-NAV200) installed on the AGV rotating tower determines the automatic positron emission and direction. The experiment is carried out on a rectangular path and shows that the AGV can follow the path to reach the target position, with an acceptable position error of less than 5% [8]. The four-wheel mobile AGV structure with the fuzzy PID controller was studied by Liu et al. In the simulation surrounding for tracing target, and as a result, they mentioned the AGV fast performance with the stability anti-interference in trajectory task [9]. Parikh et al. used Arduino and a DC motor as the main part of the AGV robot and simulated them in MATLAB Simulink. The AGV has four wheels, is driven by a DC motor, and uses infrared sensors for tracking. Then, the simulated model analyzed the speed of the AGV with the P, PI, and PID controller system, which is tuned by the Ziegler–Nicholas algorithm on a straight path to find an AGV system that can run at a uniform speed. They investigated the AGV response over the PID parameters; they found that the overshoot of the P controller is small, but the steady-state error is high and needs more time to stabilize. In the PI controller, the AGV is unstable, and with the PID controller, It can normally work with the ignorable overshoot. They concluded that the ZN algorithm and PID controller is more suitable [10]. AGV trajectory tracking control method for AGV was studied by Chen et al. The results of the AGV platform consist of two independently driven wheels and one driven wheel. Then, if the AGV needs to run in reverse, the wheels rotate the AGV's coordinate system 180 degrees, and then the tail's and head's positions will be changed. The experiments conducted on sinusoids and paths consisting of segments and arcs found their proposed control method feasible [11]. Li et al., based on the Four-Wheel Independent Steering and Four-Wheel Independent Driving (4WIS-4WID) vehicle model, proposed a system. Their study consisted of two controllers of four-wheel PID steering and a four-wheel Sliding Mode Controller (SMC) driving for driving and longitudinal motion control. The research shows that a four-wheel SMC steering controller and four-wheel combined yaw rate and longitudinal velocity SMC driving controller can achieve most of the control objectives. The simulation result indicated that the four-wheel SMC steering controller, four-wheel combined yaw rate, and longitudinal velocity SMC driving controller are most suitable for 4WID-4WIS vehicles [12]. Zhifu et al. proposed a dynamic torque distribution method for 8x8-wheel motor-driven vehicles to increase the algorithm's speed and the vehicle's response speed to complex road conditions. The vehicle uses the PID control method to track the longitudinal speed and yaw angle. The designed framework meant based on the direct yaw moment control method. Analyze the expected acceleration and expected yaw according to the driver's accelerator pedal and steering wheel signals. The vehicle stability is promised in the steering tracking process Through simulation experiments on the MATLAB/Simulink platform [13]. Setiawan et al. designed four-wheel independent steering (4-WIS) automatic guided vehicle and proposed a trajectory tracking controller based on the back-stepping method. They modelled the 4-WIS AGV by simplifying the ordinary four-wheeled vehicle model to a two-wheeled vehicle model with centreline wheels. Through simulation experiments, they found that their trajectory tracking controller can very well track sharp-edged straight and circular trajectories [14]. Richard and Gang hired the Programmable Logical Controller (PLC) and Radio Frequency Identification RFID technology in AGV to control and improve the speed of data flow. They use PID controllers for designing and using the Ziegler-Nichols method in Matlab with SISOTOOL to tune PID parameters based on critical gain and critical period [15].

Two-wheel differential AGV and PID controllers were investigated by Tang et al. This study is merged with MATLAB/Simulink as the simulation tool and takes the estimated deviation as input and the difference between the rotational speed of the left and right driving wheels as the output. After tuning the PID parameters and investigating the effect of parameters, the result shows that the designed PID controller can make the AGV perform well in the straight and arc paths [16]. The physical and simulated models are compared by Papelis and Sakioti to evaluate the fidelity of the simulation model and the influence of the motor model sensor model. They used the Stingray robot kit from Parallax and performed simulations on the Autonomous Robotics Modeling & Simulation Environment (ARMSE) platform. The line-following robot turns by the return values of 8 infrared sensors. Even though the result shows the robot's performance to follow the line, there are differences between the simulation and physical models [17]. Zhou et al. designed a fuzzy PID controller for AGV to allow the AGV to operate stably for a long time. The AGV system consists of an STM32F207 microcontroller, gray sensor, ultrasonic sensor, infrared sensor, camera, wireless module, and other peripheral devices. Due to the system complexity, they address using the Simulink to simulate a fuzzy PID control system to avoid the influence of parameter changes and external environmental interference. Then, they reported that the AGV can adaptively modify the control parameters to achieve trajectory tracking better. Experiments have found that the fuzzy PID algorithm is more stable than the traditional PID algorithm [18]. Lego Mindstorms components and Matlab Simulink were used to simulate the AGV with the simple digital control algorithm to realize the tracking function by Abdulhamid and Mutheke. The robot uses a Lego EV3 motor to drive two front wheels and a robot-supported by two rear wheels. A colour sensor is installed in the front of the robot to identify the line. They found that a robot without a controller has many swings in a straight line, which consumes energy and reduces speed, so the efficiency of this robot in a straight line is very low. Therefore, they use a PID controller. To obtain the required control effect, they tested multiple sets of *K*_*p*_, *K*_*i*_, and *K*_*d*_ parameters, and finally, they summarized the effects of the three parameters: if the value of *K*_*p*_ is increased, the rise time and steady-state error is decreased, the overshoot will be increased, and the setting time will be small changed. If the value of *K*_*i*_ increases, the rise time decreases, and the overshoot and settling time is increased. If the value of *Kd* is increased, the rise time will be a small change, the overshoot will be decreased, the setting time will be increased, and the steady-state error does not change [19]. Xu et al. proposed a hybrid control strategy that combines the yaw angle stability control of the two drive modules with the real-time algorithm to solve poor accuracy and difficulty in the arc trajectory tracking process. They divided the arc trajectory into three segments: the start, end, and arc segments. The AGV and the two differential modules were used in the arc tracking segment. The AGV assembly consists of a laser navigator, a vision sensor, and two differential modules. The front and rear differential modules include two servo drives and two drive wheels. The angular velocity constraints between the four driving wheels establish the speed coordination conditions of the four driving wheels. The trajectory and expected path are simulated by MATLAB simulation, and it is founded that the hybrid control strategy combining the yaw angle stability control module and the real-time correction algorithm has a faster response speed. The AGV can run smoothly on an arc orbit with a specified radius but is adjusted in real-time. Its flexibility is poor, and it is necessary to optimize the algorithm to achieve higher accuracy of AGV tracking in the arc trajectory [20]. The Robust AGV path tracking control strategy was pointed by Wu et al. to improve tracking accuracy and stability. They simplified the complex path tracking problem to a simple yaw angle tracking problem. The research introduces the strategy based on nonsingular terminal sliding mode (NTSM) and active disturbance rejection control (ADRC). It uses an extended state observer to estimate and compensate for the unmodelled dynamics of external disturbances of the system in real-time. During the research, the CarSim-Simulink simulation and results show that the proposed control strategy can ensure vehicle stability and quickly and accurately follow the reference path [21]. Pliego-Jiménez et al. proposed an inverse dynamics controller to control the tracking of a two-wheel differential drive vehicle. The controller is proposed to compensate for the nonlinear problems that occur during the motor action. The assembly consists of one caster and two DC motor-driven wheels that were simulated with the inverse dynamic controller in MATLAB/Simulink and were compared with the conventional PID controller. Two paths in the form of circular and straight are assumed as the reference path. The controller controls the tracking trajectory by minimizing the position and angle errors. The report shows that the proposed controller can reduce the error faster than the traditional PID controller [22]. Zhou et al. and colleagues designed a 16-wheel hard AGV system equipped with magnetic tape navigation to track the line. They simplified the 16-wheel heavy AGV into a two-wheel AGV by adjusting the navigation system location based on the Ackerman command principle. MATLAB/Simulink was used to simulate and test the path consisting of a straight line to a curve and a curved line to a straight line. They found that the following system design has an acceptable effect on plain, curved, and mixed trajectories [23]. Song proposed a trajectory tracking controller for AGV with time-varying state feedback. They used straight paths and circular curve patterns for experiments to check the controller's performance. The experiment aimed to compare the predicted trajectory by the controller and the actual one on the simulation platform. They reported the successful simulation and mentioned that different controller parameters could affect the stable response time and steady-state errors. Then, different controller parameters need to be selected according to the trajectory change to achieve a better control effect [24]. High-precision continuous attitude adjustment for AGV path tracking issues are investigated by Weng et al. This research proposed an improved model predictive control with a state classification model (SCM) and smooth transition (ST) strategy, namely SCMPC-ST. The Numerical simulation was carried out on MATLAB to compare the integral separation PID (IS-PID), standard SCMPC, and SCMPC-ST under the same resolution. They found that it is more effective to adjust the relationship between deviations in some cases than to eliminating them directly [25]. Vartika et al. investigated the two Ziegler–Nichols and genetic algorithm (GA) methods to adjust the parameters of *K*_*p*_, *K*_*i*_, and *K*_*d*_, characteristics. Besides, they compared the rise time and range overshoot characteristics of traditional PID controllers and fuzzy-based PID controllers. The research specified that to tune the PID parameters compared with the traditional ZN method, the adaptive intelligent PID controller, along with the Fuzzy logic Controller (FLC) and Genetic Algorithm (GA), can ensure improved performance parameters while tuning the parameters of the PID controller [26]. As the survey shows, the most cost-effective approach to solving this issue would be a simulation, which assists scholars in better studying AGVs and their efficiency, mainly PID tuning, to improve the movement and control. Although various control methods have been proposed, the study shows that PID controllers have been used as a basic reference for evaluating and expanding other proposed controllers. As the survey shows, PID controllers are well known in various robot applications, and researchers have tried to find the best values for these controller parameters by various methods, like meta-heuristic methods in different robot fields [27–32]. Also, in most of the reported research studies, the assumed values in the modelling section are not real, and the detail of the simulation process for the used AGV is not described from one side; on the other side, few validations on different paths shape of the proposed model are investigated. The present research has been done to present. The rest of this article is structured as bellows: in part two, the structure and parts of AGV with the mathematical equation and modelling in Matlab software are described. Then, the PID tuning method based on Ziegler–Nichols and empirical methods with the actual size and specification of the designed AGV hired on the model, and the robot performance was analyzed with the various specific path. Then, in Section 3, the principle of two metaheuristic algorithms of Particle Swarm Optimization (PSO) and Beetle Antennae Searching (BAS) is initially described and then used to optimize the PID values. Afterwards, the robot's performance was investigated over the different path shapes and angular and tangential velocities. After that, the CoppeliaSim (VREP) simulator was used to overcome the real environment test limitation, and the robot's performance over one more path along with the previous one was analyzed. Section four was mentioned as the last part for the paper's conclusion. The paper's main contribution can be summarized as follows: (1) proposing the simple and effective model for a two-wheel AGV robot along with four free wheels in detail by using the Simulink and validating the extracted values of the model with Vrep in the trajectory mission. (2) Using the real value and dimension of the AGV robot against most of the previous reported research papers and showing the real change to tune the PID controller. (3) Compare the experiment and test the robot's performance in five paths with different shapes (Circle, Ellipse, Spiral, 8 Shape, and special path) with around 8-meter lengths. (4) Introducing and comparing the empirical PID method and two famous meta-heuristic Algorithms, PSO and BAS, with the PID tuning. This paper is the extension of our conference paper “Simulation study and PID Tune of Automated Guided Vehicles (AGV),” which has been accepted and presented by the IEEE International Conference on Computational Intelligence and Virtual Environments for Measurement Systems and Applications (CIVEMSA), 2021 [33].

## 2. Automated Guided Vehicle (AGV) Robot's Construction

**Figure 1:**
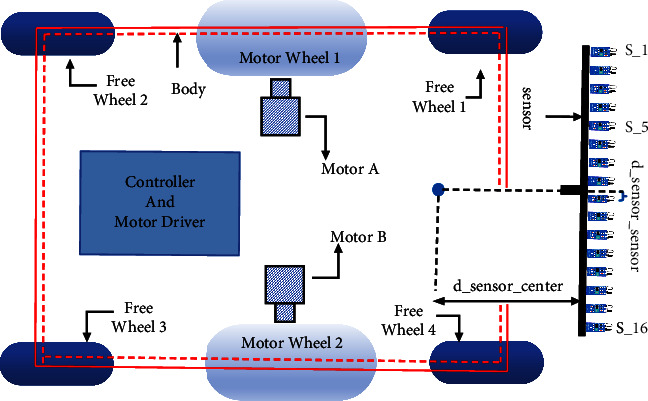
AGV vehicle top view with the sensors, actuator, and controller.

In short, the AGV robots can be defined as systems consisting of components such as the main body, motor driver (actuators), navigation and anticollisions, processor, communication systems, and batteries (Figure 1) of all the above components; the four main parts of the framework, the sensor, the actuator (motor and driver), the controller, shown in Figure 1, are particularly important.

**Figure 2:**
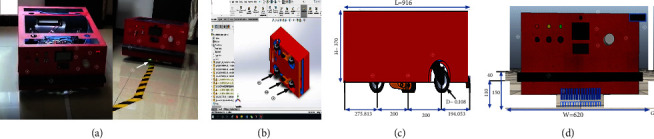
The designed AGV from different views and dimensions of AGV parts 1: battery charger, 2. alarm 3. LCD, 4: avoidance system 5: ultrasonic sensor, 6: stop/start and controlling switch set II, 8: front bumper sensor, 9: magnetic sensors,10: front freewheel, 11: brushless motor, 12: backside wheel, (a) the real designed AGV, (b) the bottom side CAD design (c) right side view CAD design (d) front side view CAD design.

Figure 1 shows the AGV platform with two motors (usually DC motors) that act as actuator devices to move and carry the body load. In addition, to compensation, the body wheels and freewheels are often used for balancing the body weights. In line with the research of this paper, the real AGV designed system has six wheels, two differential drive wheels which are located in the middle, and four passive wheels are placed in the front and back of the vehicle. These four passive wheels are responsible for maintaining the horizontal balance of the vehicle. In AGV robots, the number of freewheels based on the motor position can be two or four, and it should be remarked that based on null action to move the body, it does not affect the dynamic equation of the whole system, and it can be considered as a two-wheel robot [33]. The number of freewheels in AGV robots can be specified as two or four, depending on the location of the engine in the body. It is important to note that due to the null action for the body movement, the number of freewheels does not change the dynamic equation of the whole system. Therefore, the assumed AGV in this research with the four freewheels can be considered a two-wheeled robot [34].  Figure 2 shows the designed AGV with all system components.

**Figure 3:**
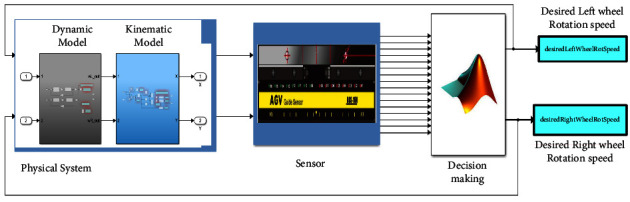
The proposed AGV model system.

As shown in Figure 2, the designed vehicle assembly consists of 16 magnetic sensors packed in one module, which are responsible for detecting the trajectory. There are three types of collision avoidance sensors (ultrasonic, PIX, and two bumper sensors at the front and rear). LCD and controlling the switch. The vehicle is 0.916 meters long, 0.62 meters wide, and 0.37 meters high, with a wheel diameter of 0.108 meters. For the designed system, Arduino Mega2560 is chosen as the main controller. As stated before, the purpose of this study is to simulate the designed AGV robot in Matlab software and validate the performance of the robot through tracking the various paths. With this aim, the main parts of the AGV can be revealed in Matlab Simulink, as shown in Figure 3.

As shown in Figure 3, in order to simulate the AGV robot, the platform can be separated into three key modules named: the physical system, sensors, and decision-making parts, which is described as follows.

### 2.1. AGV Physical System Section

According to the assumed model and structure, the dual-wheeled robot dynamic model and its related parameters were selected. Therefore, based on the mentioned assumption, the physical system includes an inverse dynamic and kinematic model, which is defined below.

#### 2.1.1. The AGV Dynamic Model

**Figure 4:**
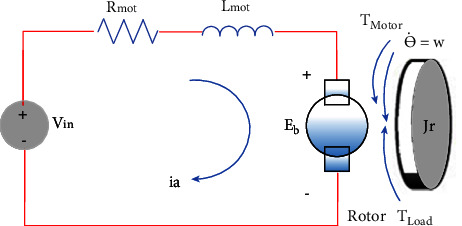
The dc motor schematic [13].

As stated before, the system assumed based on the dual-wheeled differential robot is equipped with DC motors on the left and right sides of the body. Then, as the first step, each motor should simulate and controlled (Figure 4).

Figure 4 shows the electrical equivalent circuit of DC motor, *i*_*a*_ shows the current of the armature, *R*_mot_ and *L*_mot_, electric inductance and resistance of armature, *E*_*a*_ voltage of armature terminal, *E*_*b*_ the value of back emf, *ω*_*m*_ the speed of the motor (rad/sec), *T* the torque of motor, *J*_*m*_ the friction of rotational viscous inertia. For the motor part, the back emf (*E*_*b*_) and torque (*T*) parameters can be obtained from equation (1) [35].

(1) $$\begin{array}{c} T=K_{t}∙ia\text{,}\\ E_{b}=K_{e}∙\dot{Ɵ}=K_{e}∙\omega \text{.} \end{array}$$

Equation (1) shows the dependence of engine torque (*T*) on the motor torque constant (*K*_*t*_) as the constant parameter and the armature current(*i*). and the relation of *E*_*b*_ (back emf, electromagnetic force) to the electromotive force constant (*K*_*e*_) and rotational velocity(*ω*). Considering the SI units since the motor torque is equal to the back emf and a constant value(*K*_*t*_=*K*_*e*_=*K*). Then equation (2), with respect to Newton's law and Kirchhoff's law assuming equation (1), is represented as follows:

(2) $$\begin{array}{c} J\ddot{Ɵ\;}+b\dot{Ɵ}=K∙i_{a}\text{,}\\ {BR}_{mot}i_{a}{+L}_{mot}\frac{di_{a}}{dt}=V_{in}-E_{b}\text{.} \end{array}$$

Equation (2) shows the mechanical and electrical equation of the motor. In equation (2), *J* is the moment of inertia of the rotor, *b* motor viscous friction constant, *K* signifies both the motor torque constant and the back emf constant. The input voltage (*V*_in_) and the back emf (Eb), *R*_mot,_ and *L*_mot_ represent electric resistance and inductance, respectively, as indicated in Figure 4. In order to calculate the *ω* as the motor angular velocity from the mechanical equation part, considering the $\dot{Ɵ}=\omega \;/\;and\;\ddot{Ɵ}=\dot{\omega }$ equation (2) can be rewritten as follows:

(3) $$\begin{array}{c} J\dot{\omega }+b\omega =T\;then\;\omega =\int \frac{1}{J}\left(T-b\omega \right)\text{.} \end{array}$$

Also, equation (4) can be used as an electrical part to calculate the armature current (*i*_*a*_).

(4) $$\begin{array}{c} \frac{di_{a}}{dt}=\frac{1}{L}\left({V_{in}-E_{b}-R}_{mot}i_{a}\right)then\;i_{a}=\int \frac{di_{a}}{dt}\text{.} \end{array}$$

The mentioned equations (3) and (4) can be directly hired for the motor simulation section in Simulink to calculate the armature current and angular velocity of the motor [35].

#### 2.1.2. Transfer Function of DC Motor Dynamic Model

Equation (2) is expressed with the terms of S and using Laplace transform as equations (5) and (6)

(5) $$\begin{array}{c} s\left(Js+b\right)Ɵ\left(s\right)=KI\left(s\right)\text{,} \end{array}$$

(6) $$\begin{array}{c} \left(Ls+R\right)I\left(s\right)=V_{in}\left(s\right)-KsƟ\left(s\right)\text{.} \end{array}$$

In equation (7), after eliminating *I*(*s*), the open-loop transfer function of the permanent magnet DC motor is obtained. In this transfer function, the voltage *V*_in_ as the input, and rotational speed is considered as the output. This part refers to the dynamic model of both sides of the robot motors.

(7) $$\begin{array}{c} P\left(s\right)=\frac{\theta \left(s\right)}{V_{in}\left(s\right)}\\ =\frac{K}{\left(Js+b\right)\left(Ls+R\right)+K_{t}K_{r}}\left(\frac{rad/\text{Sec}}{v}\right)\text{,} \end{array}$$

where *J* stands for rotational inertia, *b* denotes viscous friction coefficient, *L* represents the armature inductance, and *R* stands for armature resistance. Lastly, by considering the DC motor controlled by the armature parameters, a dynamic model can be extracted according to equations (8)–(10).

(8) $$\begin{array}{c} I_{a}\left(s\right)=\left[\frac{1}{L\cdot s+R_{a}}\right]\left[E_{a}\left(s\right)-E_{b}\left(s\right)\right]\text{,} \end{array}$$

(9) $$\begin{array}{c} T\left(s\right)=K_{T}\cdot I_{a}\left(s\right)\text{,} \end{array}$$

(10) $$\begin{array}{c} \Omega_{m}\left(s\right)=\left[\frac{1}{J_{m}\cdot s+B_{m}}\right]\cdot T\left(s\right)\text{.} \end{array}$$

**Figure 5:**
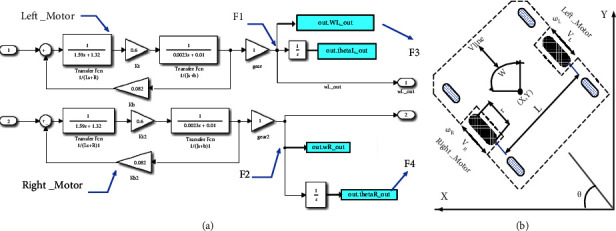
AGV system model, (a) dynamic model part in simulink, (b) parameters of kinematics.

According to the aforementioned equations and all parameters of the motor summarized in Table 1. The Simulink block diagram for this section can be derived as shown in Figure 5(a). It should be noted that in addition to the mentioned parameters, the values for other parameters like voltage of armature terminal (*E*_*a*_), the value of back emf (*E*_*b*_), speed of the motor (rad/sec) (*ω*_*m*_), and motor torque (*T*) should be calculated by a motor with respect to the suggested model.

Table 1 shows the actual values of the dc motor parameters (four-inch brushless electric wheel hub motor, 24 V DC, 280 rpm) based on the values obtained from the manufacturer's laboratory [36]. As illustrated in Figure 2, two numbers of this motor are assembled on the design AGV system.

#### 2.1.3. The AGV Kinematic Model

The next section for the physical system includes a kinematic model for the robot. In this section, the angular velocity of the left and right wheels (*ω*_*L*_, *ω*_*R*_) in terms of radians per second (rad/s) is considered as the input and the *V*_lin_ linear velocity in terms of meters per second (m/s) and *ω* the angular velocity of the robot in terms of radians per second (rad/s) assumed as the output. Based on the dual wheel model [3], as shown in Figure 5(b), *R* and *L* represent the radius of the robot wheels and the distance between the two wheels, which are connected on both sides of the robot frame. In the mentioned model, forward and reverse kinematics are inspected. The Forward kinematics (equation (11)) means the *x* and *y* coordinates along with orientation angles, which can be calculated from the length and angles of the robot and Inverse kinematics (equation (12)) means calculating the angles required to obtain the position of *x*, *y*, and the desired orientation. In kinematics, the angular velocity of left (*ω*_*L*_) and right (*ω*_*R* _) of robot wheels consider as input, and the plane coordinates (*x*, *y*) along with the heading direction of the robot *θ* as output. The equation for the forward and inverse kinematics can be shown as follows:

(11) $$\begin{array}{c} V_{lin}=\frac{r}{2}\left(\omega_{R}+\omega_{L}\right)\text{,}\\ \omega =\frac{r}{L}\left(\omega_{R}-\omega_{L}\right)\text{,} \end{array}$$

(12) $$\begin{array}{c} \omega_{L}=\frac{1}{R}\left(V_{lin}-\frac{\omega L}{2}\right)\text{,}\\ \omega_{R}=\frac{1}{R}\left(V_{lin}+\frac{\omega L}{2}\right)\text{.} \end{array}$$

In equations (11) and (12), the (*V*_lin_) stands for the robot's linear velocity, *r* stands for the wheel radius, *L* stands for the distance between the two sides of wheels, (*ω*) the angular velocity, *ω*_*L*_ and *ω*_*R*_ stands for the angular velocity of the right and left wheel, respectively. Based on the real value of the designed AGV prototype, it should be noticed that *r* is 0.054 meters, and *L* is 0.62 meters. To show the mentioned equation in the simulation program, the position and direction of the robot are required. Therefore, the velocities of both sides of the wheels (*ω*_*L*_, *ω*_*R*_) are fed into the inverse kinematics model to calculate the coordinate of the robot (*x* and *y*) and the direction angle (*θ*). Furthermore, the robot's coordinate can be derived from these equations by integral and trigonometric functions, as shown in the following equation:

(13) $$\begin{array}{c} x=\int \left(\omega_{L}+\omega_{R}\right)*\frac{r}{2}*\;\cos\;\theta \text{,}\\ y=\int \left(\omega_{L}+\omega_{R}\right)*\frac{r}{2}*\;\sin\;\theta \text{,}\\ \theta =\int \left(\left(\omega_{L}+\omega_{R}\right)*\frac{r}{L}\right)\text{.} \end{array}$$

**Figure 6:**
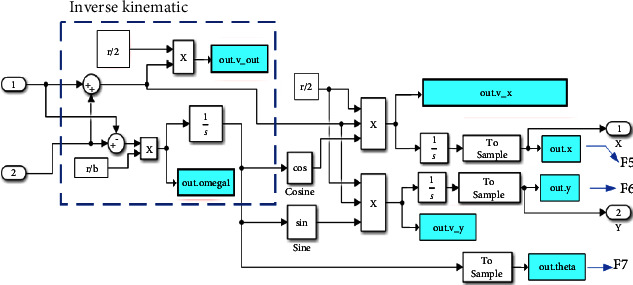
The robot coordinates *X*, *Y* and direction angle *θ* in the kinematics model.

Finally, concerning equation (13), the Simulink model is presented in Figure 6.

The parameters and formulas for the proposed models, as shown in Figures 3 and 4, are summarized in Table 2 and labelled from equations (14) to (22).

### 2.2. Sensor Section

The sensor part is the second basic AGV block. This part carried out the functionality of detecting the reference line. In the real platform, an array of magnetic sensors are installed in front of the vehicle with a working principle similar to the IR sensor, as described in our previous paper [33]. For this sensor, magnetic tape is used as the line. Then, when a sensor is in front of a magnetic tape, it shows the maximum values and will be detected by the robot's magnetic tape.

**Figure 7:**
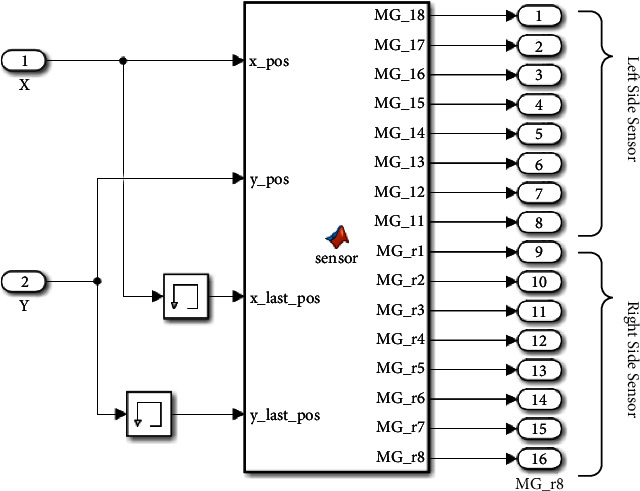
Magnetic sensor value section in simulation.

Conversely, the tape hardly reflects when the sensor is away from the magnet tape, so the sensor would not be triggered. In the simulation program, the 16 sensors are simulated by calculating the position of every magnetic sensor and comparing it with the position of the reference path. The current robot position (*x*_pos, *y*_pos) and the robot position of the last simulation frame (*x*_last_pos, *y*_last_pos) are fed into the Matlab function block, and the states of every magnetic sensor are outputs of the Matlab function block (Figure 7).

## 3. Decision-Making Component

**Figure 8:**
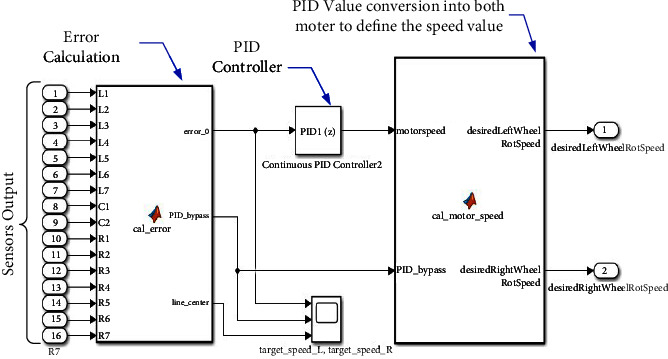
The decision-making section.

This section consists of the error calculation and the PID controller, as shown in Figure 8. The motor's direction decides based on sensor reading and PID parameters in this part.

The error calculation part has the duty to read the values of 16 sensors, and then according to the sensor values, the relevant error is extracted and entered into the PID section. As the Classical controller, the PID controller works based on the error feedback with the general equation (14) [16].

(14) $$\begin{array}{c} U\left(t\right)=K_{p}e\left(t\right)+K_{i}\;\frac{de\left(t\right)}{dt}+K_{d}\int e\left(t\right)dt\text{.} \end{array}$$

Equation (14) shows that the controller works based on three gain parameters of *K*_*p*_(proportional), *K*_*i*_(integral), *K*_*d*_(de rivation)in de xs [10]. Then, the proportional, integral, and derivative indexes are used to generate the corresponding PWM. The generated PWM commands and determined the robot speed.

In other words, the amount of error by the magnetic sensor array modes in the PID controller leads to increased and decreased speed of the robot motors. For example, based on sensor reading in a straight or winding path, the robot's speed increases or decreases to adjust the steering angle for tracking the path, as shown in Code 1.

As described in the main controller section, The PID controller is equipped to adjust the difference of the speed of both sides of the robot; thus, the robot will be able to move along the path. Its input value (Figure 8) is the error between the robot's current direction and the path's target direction, which is calculated based on the Magnetic sensor values. As shown in Code 1, the robot follows the path after extracting error values based on the sensor reading. Then, the error values were assigned as −14 to −1, 0, and +1 to +14 for left, middle, and right sensors, respectively. Then, the PID value is calculated to specify the robot's speed. When none of the sensors are triggered, which means that sensors are not facing the reference path, the PID controller is bypassed, and the output value of both sides of the wheels is set as a constant so that the robot will keep moving forward. Each sensor has a coefficient number that depends on the position of the sensor. The left sensors have negative coefficients, while the right sensors have positive coefficients. The error value is the summation of the coefficients of all the triggered sensors. Finally, the motor's voltage at the left wheel is set as a constant added by the PID output value, while the voltage of the right side is set as a constant subtracted by the PID output value.

### 3.1. PID Controller Tuning

As mentioned before, the PID controller is used to avoid oscillation and help the robot pass the path smoothly [37]. It also increases the system's stability. The PID is known as the standard traditional controlling method, and MATLAB Simulink has provided a specific tool to help design and tuning PID controller processes for wide applications. The previous research paper shows the PID controller's effect and the Ziegler–Nichols method [38] for tuning missions. As it mentioned before [33], to extract the PID loop parameters, the 1^st^ Ziegler–Nichols method (Z-N) [39] can be applied on the AGV system open-loop step response. According to the AGV model, the obtained values for the PID controller parameter after applying the 1^st^ Ziegler–Nichols method includes *K*_*p*_ = 3.263175, *K*_*i*_ = 43.677826, *K*_*d*_ = 0.060948.  Table 3 shows the step response before and after adjusting the PID parameters.

### 3.2. PID Controller Testing and Empirical PID (E_PID) Tune

As stated, the PID value directly affects the path tracking and performance of the AGV robot; then, to check the PID tuning, the calculated value is inserted in the model, and the AGV model performance is investigated. In order to validate the parameter, in this step, all mentioned blocks are connected as a closed-loop along with the real value of motor, vehicle size, and PID controller parameters which executes once every step (0.01 seconds in our configuration). Then, the AGV performance was tested in the various paths. The parameter validation has been carried out based on the compatibility of the line-tracking function under various conditions, and four experimental paths with different shapes are designed. This validation examines the amount of line tracking in four different directions [35], as shown in Table 4.

**Figure 9:**
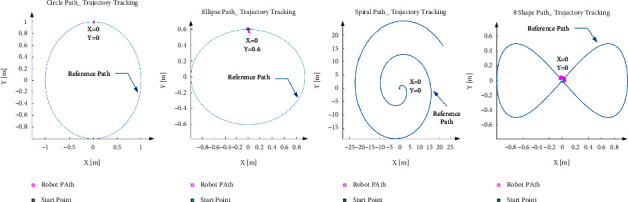
AGV model path trajectory tracking performance with *Z*-*N* values over the different paths.

To start the experiment, for each path, the robot is placed in the setpoint, and the head direction is positioned parallel to the path, the PID indexes of *K*_*p*_ = 3.263175, *K*_*i*_ = 43.677826, *K*_*d*_ = 0.060948 inserted inside the model. This process was repeated for each path at the same time, and the extracted result about position and speed were logged and plotted. The robot's movement against all paths is shown in Figure 9.

**Figure 10:**
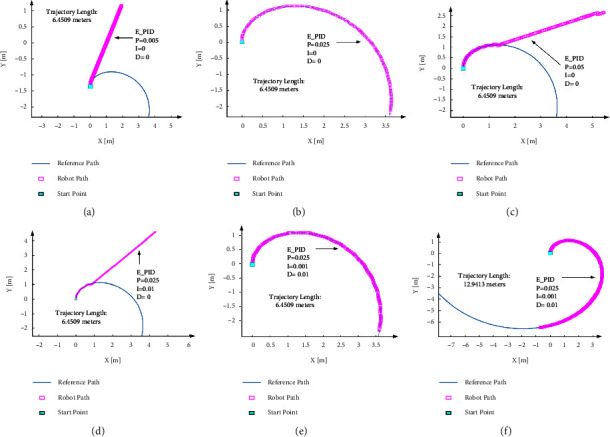
The empirical PID (E_PID) tuning method and spiral path performance.

As the result shows, the *Z*-*N* parameter against the previous effort [33] on the real platform data was unsuccessful. Then, the test based on the empirical method started to find the value that can follow the path for the robot. At first, *K*_*i*_ and *K*_*d*_ were set as 0, and many tests were carried out to find the proper *K*_*p*_ value. Based on previous experiments, the spiral path is one of the paths the robot always has the problem following. Then, the spiral path is selected to check, and robot performance is observed base on the Spiral path with a length of 6.4509 meters. The test result shows that in the case of selecting the very small value for the *K*_*p*,_ the steering angle is too small to track the path, and if the *K*_*p*_ is too big, the output value of the PID controller will diverge.  Figure 10 shows each PID value and robot performance.

As it shown initially, *K*_*p*_ is set as an intermediate value of 0.025, based on the result with *K*_*p*_ = 0.005 Figure 10(a), *K*_*p*_ = 0.025 Figure 10(b), and *K*_*p*_ = 0.05 Figure 10(c). In the next step, the *K*_*i*_ value was selected as 0.01, and the output value of the PID controller diverged Figure 10(d). Then, a smaller value of 0.001 was attempted, and the path was tracked successfully. Finally, *K*_*d*_ was set to 0.01 to reduce the oscillation, and the spiral path was tracked in Figure 10(e). After extracting the final PID value, the length of the path increased to 12.9413 meters to ensure the robot's performance Figure 10(f). The setting process of the PID values tuning attempts has been arranged based on Table 5.

**Figure 11:**
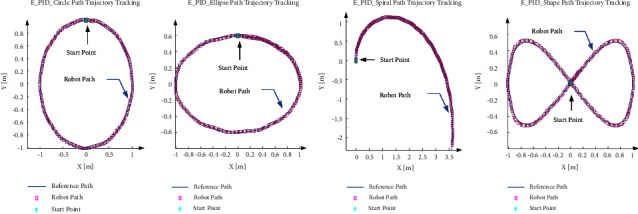
Robot achievement based on empirical PID (E_PID) index in various paths.

As Figure 10 and Table 5 shown, based on spiral path tracking, the PID controller with the value in set 5 (*K*_*p*_ = 0.025, *K*_*i*_ = 0.001, *K_d_* = 0.01) successfully follows the spiral line path. Then, to check the empirical PID value performance, the values test on all other four paths, and the tracking results are shown in Figure 11.

**Figure 12:**
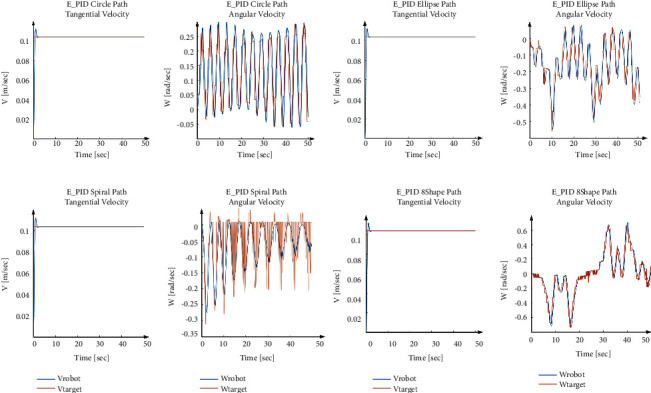
Empirical PID (E_PID) value and robot performance in different paths.

Figure 11 shows that in all four predefined paths, the robot successfully follows the path with efficient performance, and the output values follow the target values with smooth curves.  Figure 12 and Table 6 show the trajectory tracking analysis.

As it observed, among all these 4 scenarios, the tangential velocity has shown similar curves. It boosted slightly higher than the target value initially, then it approached the target value and remained stable until the end. In terms of the angular velocity of the robot and the angular velocity of both sides of the wheels, the real value was tracking the target value quite well and relatively smooth. Thus, the performance of the Empirical PID is acceptable. When running down the circle path, the angular velocity of the robot has been swinging. The angular velocity of both sides of the wheels has shown similar action that varied within a certain range. The maximum values of them are 2.6309 and 3.5055 rad/s. On the elipse path, the angular velocity of the robot also fluctuated with the angle of the ellipse path. The peak appeared when the robot arrived at the ellipse vertex. The angular velocity of the left wheels was around 4.4201 rad/s, while the target value was up to 2.6943 rad/s since the PID controller can reduce the oscillation. On the spiral path, while the robot was moving, the path became more and more straight, the angular velocity also became increasingly stable. The target velocity of both sides of the wheels has reached 3.412 rad/s and 2.4524 rad/s, respectively, during the spiral path scenario test. However, the maximum angular velocity of the two sides of the wheels were only 5.127 rad/s and 5.0059 rad/s, respectively, as the maximum value for this parameter. On the 8-shaped path, the peak of the angular velocity of the robot appeared when the robot passed through the sharp corners of the path. Even the Empirical PID values show the promise performance, but to ensure better parameters, the optimization part conducted is described in the next section.

## 4. Optimization

**Figure 13:**
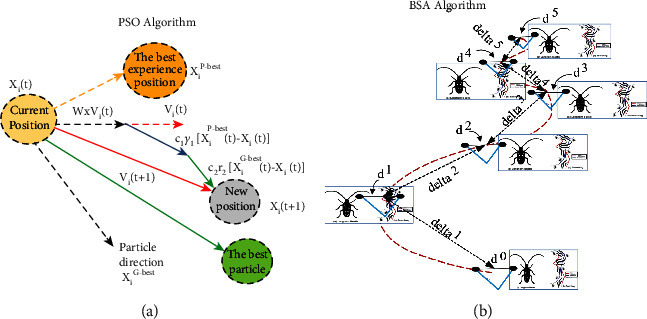
The illustration of PSO and BAS algorithms. (a) PSO algorithm. (b) BAS algorithm.

As the next step to explore the optimum values for the PID controller parameters, two metaheuristic algorithms, as shown in Figure 13, named Particle Swarm Optimization (PSO) [40] algorithm and Beetle Antennae Searching (BAS) [41], are applied. Metaheuristic algorithms have some advantages over classical methods. These algorithms are very popular because of their powerful performance in optimization problems [35]. PSO algorithm is a simple algorithm, and the number of setting parameters in this algorithm are very small. Also, this algorithm has a good performance in continuous problem spaces. This algorithm has an easy implementation, robustness to control parameters, and computational efficiency, and it is used widely by many researchers in such problems [40]. The main reason for using the BAS algorithm is being very fast and reaching the optimum point quickly. Although its accuracy is not the same as PSO, using the BAS algorithm can be useful in applications with time as a high priority [41].

Both algorithms are set to three dimensions (3-D space), and the cost function for both methods is defined as follows:

(15) $$\begin{array}{c} Cost\;function=\overset{t=t_{end}}{\underset{t=t_{start\;}}{\sum }}\sqrt{{\left(x_{tracked}\left(t\right)-x_{reference}\left(t\right)\right)}^{2}+{\left(y_{tracked}\left(t\right)-y_{reference}\left(t\right)\right)}^{2}}. \end{array}$$

Here, the *x*_tracked_ and *x*_reference_ represent the tracked and the main(reference) path in the *x* direction and *y*_tracked_, *y*_reference_ are the tracked and the main path in the *y* direction, respectively. In both methods, based on the cost function, the error is defined as the difference between the desired path and the tracked path by AGV during the simulation. The Cost function of this problem is Mean Square Error (MSE), and the optimization algorithms are used to find the optimum coefficients of the controller to minimize the error. This is a 3-D optimization problem, and the decision vector is presented in the following equation:

(16) $$\begin{array}{c} X=\left[x_{1},x_{2},x_{3}\right]. \end{array}$$

Here, *x*_1_, *x*_2_ and *x*_3_ are *K*_*p*_, *K*_*i*_ and *K*_*d*_ one-to-one and all coefficients are positive. The PSO and BAS algorithms are described briefly in the following section.

### 4.1. Particle Swarm Optimization (PSO)

The PSO algorithm was first introduced as the metaheuristic algorithm to optimize the various problem iteratively by Kennedy and Eberhard in 1995 [41] and then improved by Shi and Eberhar [42]. It is inspired by the birds behavior. In this algorithm, the founded solutions are improved regarding the cost function. PSO is known as a population-based algorithm inspired by nature and categorized as an implicit method. In PSO, a set of candidate solutions are generated inside the problem space, and then the cost function for all particles is evaluated. Then, the particles move by a vector. This vector includes three vectors (Figure 13(a)). The PSO algorithm is shown in the following pseudocode (Code 2).

As Code 2 shows, after initialization, the first part is the inertia vector, and it is alongside the current velocity vector. The second vector is the difference between the current position and the best-founded solution by that particle, and the third vector is the difference between the current position and the best-founded solution by all particles. Then, the positions of all particles are updated, and this process is repeated iteratively until the stopping criterion is met. In (17), the Mathematical formulation of the PSO algorithm is presented.

(17) $$\begin{array}{c} v_{i}=Wv_{i}+c_{1}r_{1}\left(v-x_{i}\right)+c_{2}r_{2}\left(g_{best,i}-x_{i}\right)x_{i}=x_{i}+v_{i}\text{.} \end{array}$$

In (17), *W* is the inertia weight, *v*_*i*_ and *x*_*i*_ are the velocity and current position of the i^th^ particle, *P*_best_ is the personal best of the i^th^ particle, *r*_1_, *r*_2_ as the random numbers between zero and one and *c*_1_, *c*_2_ are acceleration coefficients.

### 4.2. Beetle Antennae Searching (BAS)

The Beetle Antennae Searching (BAS) is proposed and inspired by the searching behaviour of longhorn beetles (Figure 13(b)). This algorithm imitates the function of antennae and the random walking mechanism of beetles in nature by the two main steps of detecting and searching [41]. According to the biological study, when the beetle seeks food, it does not know the food location but adjusts the foraging route according to the intensity of the food's smell. The beetle has two long antennae, and its movement is arranged based on the stronger smell on each antenna side. The subsequent Code 3 shows the complete BAS algorithm.

As it is shown in the BAS, based on assigned search space, the left and right antennae positions (*x*_*r*_ and *x*_*l*_) specified the next position of the beetle, and the position of the beetle is frequently updated to gradually reach the optimal target. Firstly, the value for the position of the beetle and the direction of the beetle antenna is initialized, and then the beetle movement direction, according to the initial value of the left and right antenna, is calculated. After that, the beetle's position and direction are updated, and the value of the left and right antenna in the current position to move the beetle in the next stage is calculated. Then, the position obtained in each iteration for three variables of *K*_*p*_, *K*_*i*_, and *K*_*d*_. Afterwards, the fitness value is calculated based on the error generated by the system after substituting that into the fitness function. The size between the fitness value and the set minimum threshold is compared. In the next step, it stops if the fitness value is greater than the threshold or the algorithm reaches the maximum number of iterations. Otherwise, it jumps back to the previous step until the stopping criterion is met, and finally, the optimum solution is shown.

### 4.3. PSO and BAS Implementation and Stopping Criteria

Generally, in the meta-Heuristic algorithms, all parameters are not exactly defined, and some settings of these algorithms are depended on the problem space and its specifications. To adjust the parameters, the algorithm has run with initial settings and based on the history of the cost function and its efficiency, the settings of the algorithm for the problem are modified. The settings of the PSO and BAS algorithms are presented in Table 7.

Table 7 shows the PSO algorithm based on the coefficient selection process in [42]. As it can be seen, the numerical value for both parameters, the number of repetitions and the size of the population, is 10. The main reason for selecting these values is that the higher population and iteration numbers only increased the calculation time without a significant improvement in the results. The values of *c*_1_, *c*_2_ are 2 and *r*_1_, *r*_2_ are random numbers between zero and one. Also, different values for the parameters of the BAS algorithm are applied, and the best results based to the minimum cost function are presented. The lower number of iterations did not lead to the minimum cost function value. Details in [41] present the process of tuning the BAS coefficients. It should be noted that in both algorithms, the number of iterative loops is considered to be 10, and the graphs are drawn result from 10 repetitions of the algorithms. Besides, generally in optimization problems, different methods are used for stopping the Optimization process. Some of the most commons are listed as follows:

- The number of iterations or the Number of Function evaluations (NFE) reaches a specific number
- The optimization time is over
- The difference between the founded solutions in each iteration and the previous level is smaller than a specific value
- The algorithm cannot find better solutions for several iterations

**Figure 14:**
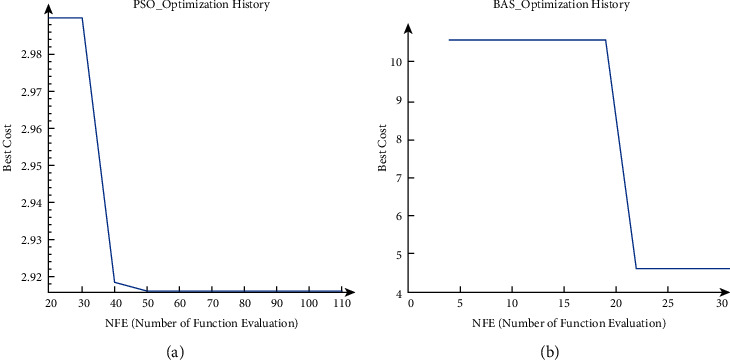
The optimization history for PSO(A) and BAS(B) algorithm. (a) PSO_optimization history. (b) BAS_optimization history.

To find the optimum solutions, both algorithms call the cost function. The Number of Function Evaluation (NFE) indicates the number of using the cost function to obtain its value based on the input values.  Figure 14 presents the history of cost function vs the number of function evaluations (NFE) for both PSO and BAS algorithms.

Considering Figure 14, it can be seen that in both figures, the value of the cost function are decreasing. In PSO, after 10 iterations, the value of the cost function is 2.918, and the value of the cost function for the BAS algorithm is 4.619.By performing the Optimization, the best-obtained coefficients for *K*_*p*_, *K*_*i*_ and *K*_*d*_ for PSO, *K_p_* = 0.02238268877, *K*_*i*_ = 0.0006907332736, *K*_*d*_ = 0.009376291668 and for BAS, *K*_*p*_ = 0.03, *K*_*i*_ = 0.0001, *K*_*d*_ = 0.02 respectively explored.

## 5. Result

The model evaluation was conducted in three-step, firstly the optimized PID parameter was tested on the model over the paths; secondly, all PID parameters(empirical, PSO, and BAS) were compared by the error as the difference between the reference line and tracked line with the histogram and correlation factors analyzation, finally to overcome the space limitation the CoppeliaSim (VREP) simulator along with four previous paths one more path named special path tested and the result compared. The mentioned steps are described as follows.

### 5.1. PSO and BAS PID Parameter Test

**Figure 15:**
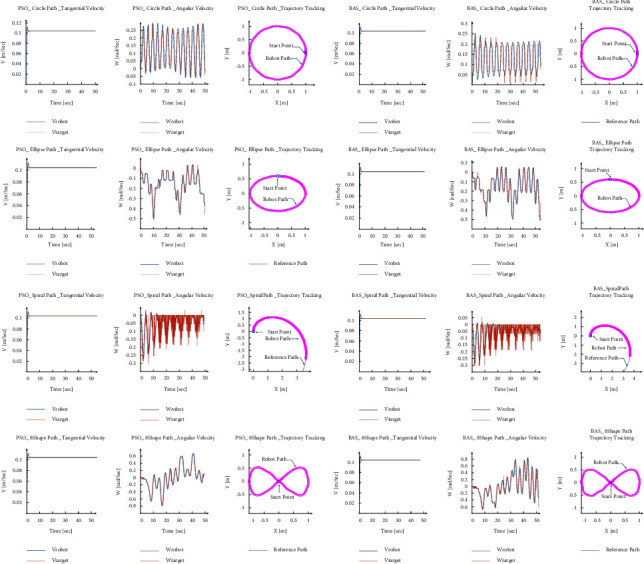
Robot performance vs PSO and BAS PID values in different paths.

In order to compare the Optimization PID value and empirical result, the explored values are fed into the simulated model. The results for each path are summarized in Table 8 and Figure 15.

As Figure 15 depicted, both values of the PID parameter for PSO and BAS algorithms tracked the path successfully. In these algorithms, the same as Empirical PID values the tangential velocity slightly overshoots in all paths and then follows the target velocity. Also, the maximum angular velocity belongs to the 8-shape path, and the least belongs to the circle path trajectory. The error is defined as the distance between the tracked and the reference paths. Then, with respect to the maximum error, except for the spiral path, BAS has less error than following the reference path.

### 5.2. PID Parameter and Histograms/Correlation of Errors

**Figure 16:**
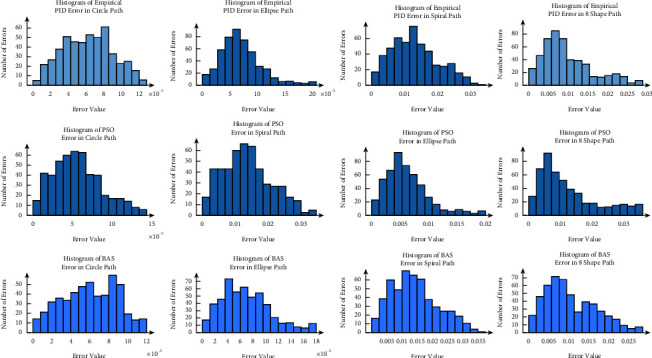
The histogram of errors.

To compare all three method PID values, Figures 16(a)–16(c) depicted the histograms of errors over all the following paths.

**Figure 17:**
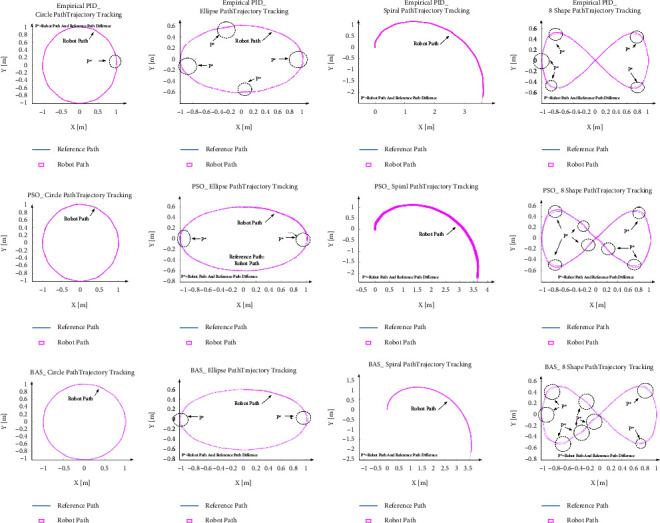
The PID values with empirical, PSO, and BAS vs robot path tracking.

It can be seen that all errors are very small and close to zero. In addition, the errors of the circle path are the smallest. Besides, Figure 16 shows that the controller can track the circular path with the best accuracy. However, even as Figure 17 shows, the number of points compared to the reference path in which the robot did not exactly follow the path shown with the *P*^*∗*^ are less and very near to the paths. Eventually, BAS values show better performance, but all three values are acceptable as the errors for other paths are small.

The Pearson correlation coefficient is an important criterion for judging the goodness of results. This coefficient measures the linear correlation between two data sets, and its value is between −1 and 1. If the correlation coefficient is 1, then it means that a linear equation can relate these two values. Considering two sets as *x*, *y*, the value of 1 for the correlation coefficient means that all data points are on a line, and as *x* is increasing, then *y* is increasing and vice versa for −1. A zero value for the correlation coefficient means that there is no linear relationship between these two sets. Equation (18) presents the mathematical formulation of the Pearson correlation coefficient.

(18) $$\begin{array}{c} r=\frac{\overset{n}{\underset{i=1}{\sum }}\left(X_{tracked,i}-\bar{X_{Tracked}}\right)\left(X_{Ref,i}-\bar{X_{Ref}}\right)}{\sqrt{\overset{n}{\underset{i=1}{\sum }}{\left(X_{tracked},i-\bar{X_{Tracked}}\right)}^{2}{\overset{n}{\underset{i=1}{\sum }}\left(X_{Ref,i}-\bar{X_{Ref}}\right)}^{2}}}. \end{array}$$

In (18), *n* is the number of data points and *X*_tracked_ and *X*_Ref_ represent the tracked and the reference path of the AGV. To evaluate the errors, the histogram of errors are depicted. It can be seen that most errors are close to zero. In Table 9, the correlation coefficients for all four paths in both *x* and *y* directions are presented.

Considering Table 9, it can be seen that for all paths, the correlation coefficients between the reference path and the tracked paths are very close to 1. Based on the statistical results, the obtained coefficients for the PID controller lead to an acceptable accuracy in tracking for the AGV.

### 5.3. Model Evaluation with CoppeliaSim (VREP)

**Figure 18:**
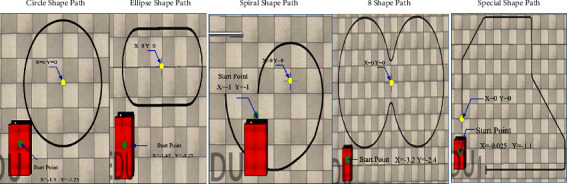
AGV robot and explored PID value vs path.

The explored PID values are tested on a real platform (Figure 2), and the explored PID values successfully followed the line but considering the size *o* robot and tested path and to overcome the environment test size, The CoppeliaSim (VREP) simulator Version 4.0.0 [43] as the most popular simulator in the robotic studies used. CoppeliaSim (VREP) was hired for three reasons. Firstly, testing the MATLAB model and evaluating extracted PID value as the main target of our research. Secondly, the Independency of designing the model to the mathematical equation and showing the real platform performance and, thirdly, extending the tracking path and adding one more path with the comparison due to the real environment limitation. With the mentioned missions, the described PID algorithm along (Code 1) with the designed AGV as per as real dimension and shape (Figure 2) implemented in CoppeliaSim (VREP) and the tracking result for five paths of circle path (7.945 meters), “ellipse shape” path (7.987 meters), spiral path (7.780 meters), “8 shapes with the 22.695 meters length, and “special shape” path (18 meters) observed and recorded result shown in Figure 18 as well as Table 10.

As it dissipated in Table 10 and Figure 18, through simulation experiments, it was found that all the extracted PID values successfully follow the path smoothly, which is in hand with the Matlab modelling section. The results show that even the robot for each test started from different origin locations in space, but the robot's movement speed was around 0.053 m/s. The robot's performance in the *V*REP environment shows that the maximum orientation for the body belongs to the robot's first touch of the path, which may happen by the right-side sensors instead of the center sensors. The result recorded shows the maximum body average degree belongs to the Special path and the least one belongs to Spiral.and the 8-shape path with the longest length takes the maximum, and the circle with the 7.945 meters has the minimum time to follow. The average of both body degrees is very near, and it shows in all the paths the maximum and minimum of degree for body degree is between -90 and 90 except in 8 shape and special path in which minimum body degrees are −32 and –0.66 in empirical PID values.

## 6. Conclusions

This paper presents the modelling and simulation of dual-wheeled AVG systems equipped with an array of 16 magnetic sensors accompanying the PID controller in MATLAB software. In this research, first, the details of the AGV model simulation, including the main parts of the body, engine and driver, processor, and sensors, along with the model construction steps, are described. As it mentioned in the survey section, most of the parameters in the previously reported paper are unreal or used with the scale. Then, the exact value of the real AGV designed to adjust the PID controller, along with the exact steps and path tracking analysis by Z-N and experimental PID methods, were investigated to build the real model. After that, two optimization methods named PSO and BAS were used to have the optimum values of PID parameters and check the robot's performance. In each experiment, the robot was put on a designed path and was instructed to move along the path. Even though the main controller selected on the real platform (ARDUINO MEGA 2560) has a data sampling limit below 1 kHz, this point is not taken into account in the system's response time during modelling and checking the response in the simulation section. Some limited tests on the real platform with the obtained PID values showed successful tracking for the platform but to evaluate the simulated model and extracted PID value, the model compared with the AGV performance in CoppeliaSim software (*V*REP software) over the mentioned path in MATLAB and the special shape path. CoppeliaSim software was selected based on more realistic performance and easy coding experience for various movement algorithms, along with its indecency to the pure mathematical AGV model.

In conclusion, according to obtained result in the simulation section, the PID controller by empirical tuning method, PSO and BAS showed a good performance to track the path in Matlab and CoppeliaSim (*V*REP) simulator. The same achievement for different path tracking missions in MATLAB and CoppeliaSim (VREP) simulator demonstrates the robot model ability and trusted values for the PID controller. The tested path shows that the tracking error of the PID controller is minimum in the circle and maximum in the spiral path. In most cases, the BAS showed better results than other methods. The results obtained from the experiments indicate the capability of the model presented in the simulation section and the similarity of the performance with the *V*rep simulator that can be useful for researchers in the field of service robots. In future work, to have a complete model, after applying all the sensors, it is suggested to check the robot model and real performance in the presence of obstacles as well as more metaheuristic optimizer controller parameters in realistic environments.

## Figures and Tables

**Algorithm 1:**
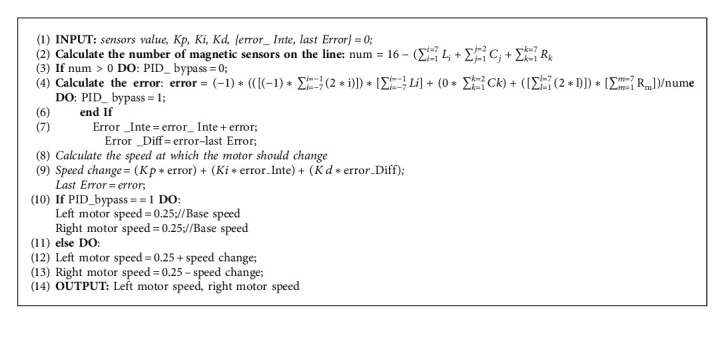
PID algorithm.

**Algorithm 2:**
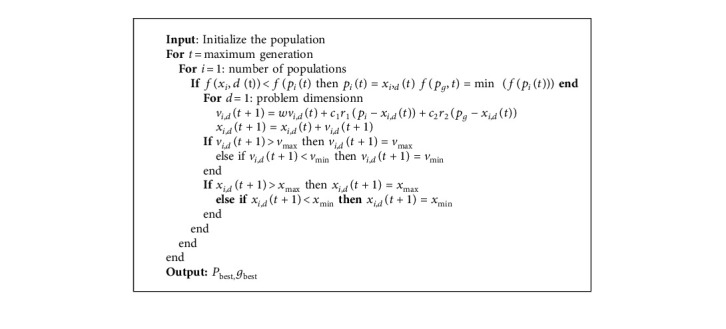
Particle swarm optimization (PSO) algorithm.

**Algorithm 3:**
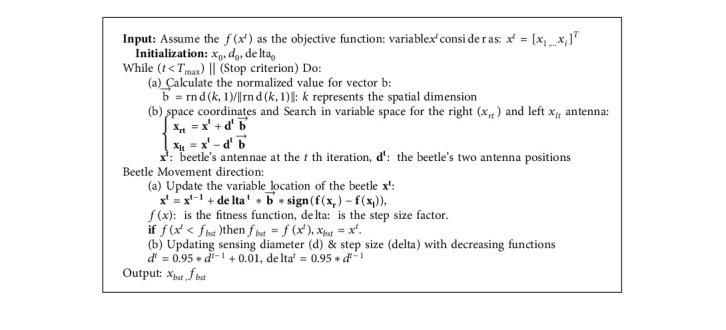
Beetle antennae searching (BAS) algorithm.

**Table 1:** The dc motor (wheel hub motor) parameter [36].

Symbol	Description	Value	Unit
*R*_*a*_	Armature resistance	1.32	Ohm
*L*_*a*_	Armature inductance	1.59	mH
*J*_*m*_	Rotational inertia	0.0023	Kg.m^2^
*K*_*e*_	Back-EMF constant	0.082	V sec/rad
*K*_*t*_	Motor torque constant	0.6	N m/A
*B*_*m*_	Viscous friction	0.01	N/rad/sec

**Table 2:** The AGV robot's physical system parameters and equations.

Angular velocity of the left wheel	F1=*ω*_*L*_	(14)
Angular velocity of the right wheel	F2=*ω*_*R*_	(15)
The velocity of the robot	*F*3=(*F*1+*F*2)*∗r*/2	(16)
Angular velocity of the robot	F4=((*F*2 − *F*1)/*r*/*b*)	(17)
Robot velocity in the *x*-axis	F5=F3 *∗* cos *θ*	(18)
Robot velocity in the *y*-axis	F6=F3 *∗* sin *θ*,	(19)
Robot position in *x*-axis	*x*=∫*F*5,	(20)
Robot position in *y*-axis	y=∫F6	(21)
The angle of the robot	*θ*=∫F4	(22)

**Table 3:** System step response.

No	Parameter (unit)	Before tuning	After tuning
1	Rise time (second)	0.018495	1.643292
2	Settling time (second)	1.465049	2.892443
3	Settling min (second)	0.270460	88.606993
4	Settling max (second)	2.075210	98.279464
5	Overshoot (%)	86.967466	0
6	Undershoot (%)	0	0
7	Peak response (%)	2.075210	98.279464
8	Peak time (%)	0.053879	8.867674

**Table 4:** The path shape and formula.

No	Path name	Path formula	Length (meters)
1	Circle shape	*x* = 0.3 × cos (0.3 × *t*); *y* = 0.3 × sin (0.3 × *t*);	7.9293
2	Ellipse shape	*x* = 0.5 × sin (0.3 × *t*); *y* = 0.3 × cos (0.3 × *t*);	7.9301
3	Spiral path	*x* = 0.1 × *t* × cos (0.3 × *t*); *y* = 0.1 × *t* × sin (0.3 × *t*);	7.9309
4	8 shape path	*x* = 0.5 × sin (0.3 × *t*); *y* = 0.5 × sin (0.3 × *t*) × cos (0.3 × *t*)	7.9282

**Table 5:** The E_PID value steps.

PID values	*P*	*I*	*D*	Performance	
Try 1	0.005	0	0	Fail	(Figure 8a)
Try 2	0.025	0	0	Tracked	(Figure 8b)
Try 3	0.05	0	0	Fail	(Figure 8c)
Try 4	0.025	0.01	0	Fail	(Figure 8d)
Try 5	0.025	0.001	0.01	Tracked	(Figure 8e)

**Table 6:** Robot performance details over the different paths.

Trajectory name/length (meter)		Circle (6.4509)	Ellipse (6.4509)	Spiral (6.451)	8 shape (6.4507)
1	Min wl (rad/s): left	0	0	0	−0.19819
2	Maxwl (rad/s): left	2.6309	4.4201	3.412	5.127
3	Minwr (rad/s): right	0	0	0	−0.31928
4	Maxwr (rad/s): right	3.5055	2.6943	2.4524	5.0059
5	Max target_speed: left	2.595	4.4923	3.5969	5.1558
6	Min target_speed: left	1.253	2.1112	2.208	−0.16424
7	Max target_speed: right	3.497	2.6388	2.542	4.9142
8	Min target_speed: right	2.155	0.2577	1.1531	−0.40584
9	Max error (mm)	0.01310	0.01927	0.03456	0.02953
10	Min error (mm)	0	0	0	0

**Table 7:** The settings of the PSO and BAS algorithm.

	PSO settings		BAS settings	
	Parameter name	Value	Parameter name	Value
1	Maximum iteration	10	Maximum iteration	10
2	*n* pop (population number)	10	*d*0	0.001
3	Inertia weight	1	*d*1	3
4	*c*_1_, *c*_2_	2	Eta	0.95
5	*r*_1_, *r*_2_	Random numbers between 0 and 1	Step length	0.8
			Eta step	0.95

**Table 8:** Robot performance details over the different path.

Trajectory length (m)		Circle (6.4509 meters)		Ellipse (6.4509 meters)		Spiral (6.451 meters)		8 shape (6.4507 meters)	
Simulated AGV performance		PSO	BAS	PSO	BAS	PSO	BAS	PSO	BAS
1	Left: minwl	0		0		0		−0.11891	0.73282
2	Left: maxwl	2.4337	2.3429	4.2874	4.2932	3.3956	3.4397	5.3677	4.9038
3	Right: minwr	0		0		0		−0.56002	−0.096143
4	Right: maxwr	3.3906	3.3332	2.5382	2.6329	2.4266	2.4649	4.9266	5.5405
5	Max error	0.0146	0.0117	0.02025	0.01794	0.03354	0.03476	0.03507	0.027823
6	Min error	0		0		0		0	
7	Left max target speed	2.4886	2.375	4.2495	4.1749	3.5207	3.5276	5.277	4.9572
8	Left min target speed	1.357	1.4933	2.2595	2.1824	2.2591	2.1575	−0.10897	−0.75791
9	Right max target speed	3.393	3.2567	2.4905	2.5676	2.4909	2.5925	4.859	5.5079
10	Right min target speed	2.2614	2.375	0.5005	0.57508	1.2293	1.2224	−0.527	−0.20722

**Table 9:** The correlation coefficients.

Path	Empirical PID		PSO		BAS	
	*X* correlation	*Y* correlation	*X* correlation	*Y* correlation	*X* correlation	*Y* correlation
Circle	0.999979	0.999976	0.999985	0.999980	0.999988	0.999987
Ellipse	0.999975	0.999938	0.999975	0.999959	0.999979	0.999966
Spiral	0.999975	0.999919	0.999976	0.999919	0.999977	0.999921
8 shape	0.999951	0.999814	0.999940	0.999612	0.999939	0.999785

**Table 10:** The *V*rep simulation performance over paths.

Path	Method	Length path (meter)	Time (second)	Origin location (meter)	Average speed (m/s)	Body change (degree)		
						Average	Max	Min
Circle	Empirical PID	7.945	147	(−1.3, −1.25)	0.054	8.397	90	−90
	PSO					8.747		
	BAS		150		0.053	8.624		

Ellipse	Empirical PID	7.987	148	(−1.45, −1.25)	0.054	9.496	90	−90
	PSO					9.562		
	BAS		151		0.053	9.630		

Spiral	Empirical PID	7.78	144	(−1, −1)	0.054	−5.854	90	−90
	PSO		146		0.053	−5.719		
	BAS		147			−5.915		

8 shape	Empirical PID	22.695	428	(−3.2, −2.4)	0.053	27.548	90	−32
	PSO					27.650		−90
	BAS					27.612		

Special	Empirical PID	18	340	(−0.025, −1.1)	0.053	39.798	90	−0.66
	PSO					39.853		−90
	BAS					39.746		

## Data Availability

The data used to support the findings of this study are available from the corresponding author upon request.
